# Association between sleep quality and cardiovascular damage in pre-dialysis patients with chronic kidney disease

**DOI:** 10.1186/1471-2369-15-131

**Published:** 2014-08-12

**Authors:** Jun Zhang, Cheng Wang, Wenyu Gong, Hui Peng, Ying Tang, Cui Cui Li, Wenbo Zhao, Zengchun Ye, Tanqi Lou

**Affiliations:** 1 Division of Nephrology, Department of Medicine, 3rd Affiliated Hospital of Sun Yat-sen University, Guangzhou 510630, Guangdong, China; 2 Division of Nephrology, Department of Medicine, 2rd Affiliated Hospital of Sun Yat-sen University, Guangzhou 510120, Guangdong, China

**Keywords:** Sleep quality, Pittsburgh Sleep Quality Index (PSQI), Chronic kidney disease, Cardiovascular damage, Left ventricular hypertrophy (LVH)

## Abstract

**Background:**

Poor sleep quality, a novel risk factor of cardiovascular diseases (CVD), is highly prevalent in patients with chronic kidney disease (CKD). The association between poor sleep quality and cardiovascular damage in patients with CKD is unclear. This study is aimed to assess the prevalence and related risk factors of sleep disturbance and determine the relationship between sleep quality and cardiovascular damage in Chinese patients with pre-dialysis CKD.

**Methods:**

A total of 427 pre-dialysis CKD patients (mean age = 39 ± 15 years, 260 male/167 female) were recruited in this study. The demographics and clinical correlates were collected. The sleep quality was measured by the Pittsburgh Sleep Quality Index (PSQI), whereas the cardiovascular damage indicators (the Early/late diastolic peak flow velocity (E/A) ratio and left ventricular mass index (LVMI)) were determined by an echocardiographic examination.

**Results:**

Of the CKD patients, 77.8% were poor sleepers as defined by a PSQI score > 5. Median estimated glomerular filtration rate (eGFR) was 69.4(15.8-110.9) ml/min/1.73 m^2^. Logistic regression analysis revealed that left ventricular hypertrophy (LVH) was independently associated with the PSQI score (OR = 1.092, 95% CI = 1.011-1.179, p = 0.025), after adjustment for age, sex and clinical systolic blood pressure, diastolic blood pressure, Phosphate, Intact parathyroid hormone (iPTH), Hemoglobin and eGFR. The linear regression analysis showed that the E/A ratios were independently associated with the PSQI score (β = -0.115, P = 0.028) after adjustment for a series of potential confounding factors.

**Conclusions:**

Poor sleep quality, which is commonly found in pre-dialysis CKD patients, is an independent factor associated with cardiovascular damage in CKD patients. Our finding implies that the association between poor sleep and CVD might be mediated by cardiac remodeling.

## Background

Sleep-related problems and disorders are common problems in end-stage renal disease (ESRD) patients [1]. Several studies have shown that the prevalence of sleep problems including sleep apnea, insomnia, periodic limb movement disorder (PLMD), restless leg syndrome and overall poor sleep quality ranges from 30 to 80% among these patients [1,2]. There is substantial evidence indicating that insufficient sleep and poor sleep quality promote the development and exacerbate the severity of three important risk factors for chronic kidney disease (CKD), namely, type 2 diabetes, hypertension and obesity [3]. Poor sleep quality has been associated with an increased risk of mortality in pre-ESRD patients [4]. Recent studies have shown that poor sleep quality was related to the progression of cardiovascular disease (CVD) [5,6]. CVD is the most common cause of death in CKD patients in almost all stages. A previous study found that poor sleep quality was associated with a higher rate of CVD in continuous ambulatory peritoneal dialysis (CAPD) patients [7]. The pathophysiological pathways between poor sleep patterns and CVD remain unclear. Relatively few studies have examined sleep problems in the considerably larger group of patients with pre-dialysis CKD, and the related factors of poor sleep quality are not clear in these populations. Recent studies reported that the prevalence of poor sleep in CKD (prior to ESRD) varies widely from 14% to as high as 85% [3]. The association between sleep quality and specific laboratory variables was inconsistent in these studies. Kurella et al. reported a significant association between eGFR and sleep quality [8], whereas other studies failed to show a relationship between renal function and sleep quality [4,9]. Further research with a larger sample size and a different study population is needed.

There is no sleep quality data on Chinese pre-dialysis CKD patients. In this study, we aimed to assess the prevalence and associated risk factors of self-reported poor sleep quality and whether self-reported poor sleep quality is associated with biomarkers of cardiovascular damage (LVH and E/A ratio) disease in Chinese CKD patients.

## Methods

The study protocol was approved by the Ethics Committee of the 3^rd^ hospital of Sun Yat-Sen University, and all of the study participants provided written informed consent before enrolling in this study.

### Patients

The patients were recruited from May 2009 to Dec 2012. The CKD patients were diagnosed using the clinical practice guidelines set by the National Kidney Foundation Disease Outcomes Quality Initiative (NKF-K/DOQI) [10]. The inclusion criteria were as follows: 1) Pre-dialysis CKD patients with a stable disease state; 2) respondent to the sleep questionnaire and able to read and write and without a communication barrier. The exclusion criteria were as follows: 1) age <18 years; 2) treatment with corticosteroids or hormones; 3) acute changes in the estimated glomerular filtration rate (eGFR) >30% in the previous 3 months; 4) pregnancy; 5) a history of abuse of drugs or alcohol; 6) night or shift work employment; 7) diagnosed sleep apnea and chronic obstructive pulmonary disease; 8) known acquired immunodeficiency syndrome (AIDS) or active infection; 9) acute cardiovascular disorders (unstable angina pectoris, heart failure, life-threatening arrhythmia, atrial fibrillation); 10) renal replacement therapy (hemodialysis, peritoneal dialysis or renal transplant); Body mass index more than 30 kg/m^2^.

A total of 498 patients were diagnosed with CKD: 10 patients were treated with corticosteroids or hormones, 5 patients were < 18 years old, 6 patients had an acute eGFR decrease, 8 patients had acute cardiovascular disorders, 9 patients had an infection, 12 patients were in renal replacement therapy and 3 patients had shift work employment, and these patients were excluded according to the exclusion criteria. Remaining patients (n=445) was recruited for the study: 18 patients could not complete the questionnaire (10 patients could not read and write or had a communication barrier; 5 patients’ questionnaires were invalid; and 3 patients refused to complete the questionnaire). Finally, 427 patients completed the questionnaire and were enrolled in the study.

### Measurement

Sleep quality was measured using the Pittsburgh Sleep Quality Index (PSQI) [11], which contains 19 items. The PSQI is a self-administered questionnaire to assess a patient’s sleep quality during the past month. From the patients’ answers, the 19 questions are grouped into seven components scores, including subjective sleep quality, sleep latency, sleep duration, sleep efficiency, sleep disturbance, use of sleep medications, and daytime dysfunction, were calculated. Each component was scored from 0 to 3 to yield a global PSQI score from 0 to 21. A high PSQI score indicates poor sleep quality. According to Buysse et al., patients with a PSQI score > 5 are conventionally defined as “poor sleepers”, whereas those with a score ≤ 5 are considered “good sleepers”. A previous study of primary insomniacs and healthy controls in community-dwelling adults reported that the cutoff score of 5 in the Chinese version PSQI has a sensitivity and specificity of 98% and 55%, respectively [12].

Depression were assessed using the short form of the Beck Depression Inventory (BDI-SF) [13], a widely used 13-item questionnaire scored on a 4-point scale, from 0 to 3, with overall scores ranging from 0 to 39. Overall scores of 0–4 indicate no depression, 5–7 indicate mild, 8–15 indicate moderate and 16 and more indicate severe depression. The BDI-SF has been found to have a good correlation with the standard 21-item BDI (r = 0.96, p = 0.001) and relates to the clinical depth-of-depression (r = 0.61) [13], with similar diagnostic efficiency [14]. We administered the Chinese version of the Beck Depression Inventory (BDI) to evaluate depression in the patients [15].

### Other data collected

The clinical data recorded included duration of CKD, gender, age, current tobacco and alcohol intake, height and weight, blood pressure (BP), 24-h urinary protein excretion, serum albumin (ALB), serum creatinine (SCR), Serum uric acid, total cholesterol (TC), triglycerides (TG) and hemoglobin (HGB). The eGFR was estimated using the Chinese abbreviated MDRD (Modification of Diet in Renal Disease) equation [16]. The 24-h urinary protein excretion was calculated from a 24 h urine collection. The Scr, TC, ALB and TG concentrations were measured by a Hitachi 7180 biochemistry autoanalyzer (Hitachi, Tokyo, Japan; reagents from Roche Diagnostics, Mannheim, Germany). The Scr levels were measured by enzymatic and traceable to the isotope dilution mass spectrometry at the time of the study.

#### Carotid ultrasonography

Carotid intima-media thickness (cIMT) was assessed by two trained investigators before study commencement. A SonoSite MicroMaxx Ultrasound System paired with a 5–10-MHz Multifrequency High-resolution linear transducer (Bothell, WA, USA) with Sono-Calc IMT software was used for taking automatic measurements of cIMT. This was achieved by averaging three measurements taken on each carotid artery (anterior, lateral and posterior directions) and measuring the distance between the leading edge of the lumen–intima interface and the leading edge of the collagenous upper layer of the adventitia using high-resolution B-mode ultrasonography.

#### Cardiac assessment

Two-dimensional echocardiography was used to assess the volume, mass, systolic function and diastolic function of the left ventricle. The left ventricular mass was calculated using the Devereux method [17]. The left ventricular mass index (LVMI) was obtained by calculating the left ventricular mass to the height^2.7^[18]. The males with a LVMI of >49 g/m^2.7^ and the females with a LVMI of > 45 g/m^2.7^ were defined as having left ventricular hypertrophy (LVH) [19]. The left ventricular systolic function was assessed by the left ventricular ejection fraction (LVEF). Diastolic function was assessed by recording mitral flow with standard pulsed Doppler technique [20], and measurements of early diastolic peak flow velocity (E), late diastolic peak flow velocity (A) and the ratio of early to late flow velocity peaks (E/A ratio).

### Statistical analyses

The data were analyzed using SPSS software, version 15.0 (SPSS, Chicago, IL, USA). The descriptive statistics are presented as percentages for the discrete variables and as the means (standard deviation) for the continuous variables. The non-parametric variables are expressed as the median and interquartile range. The comparisons of the continuous variables between the groups were tested by Student’s t-test or the non-parametric test. The differences among the categorical variables were analyzed using the χ^2^ test or the two-tailed Fisher’s exact test, as appropriate.

In the univariate analyses, a linear regression model was employed to examine the association between the sleep quality scores (the dependent variable) and the independent variables, including duration of CKD, diabetes (yes/no), current smoker (yes/no), alcohol intake (yes/no), BMI, BDI-SF score, clinic BP and clinical variables. A multiple linear regression (stepwise method) was performed to evaluate the relationship between the sleep quality scores and those independent variables with a potential association (crude p < 0.15 in univariate analyses) with a dependent variable in a univariate linear regression.

Univariate and multivariate logistic regression analyses were used to explore the independent associations of LVH (LVH vs. no LVH) with sleep quality scores. A univariate and multiple linear regression model was also employed to examine the association between the E/A ratio (dependent variable) and the sleep quality scores. All potential association variables (crude p < 0.15 in univariate analyses) and BDI score were tested further in multivariate regression analyses. All of the values are two-tailed, and the significance is defined as p < 0.05. Age and sex were forced to be included in the regression models for all of the regression analyses.

## Results

### Sample characteristics

The mean age of the subjects was 39 ± 15 years (range 18–70 years). Median estimated glomerular filtration rate (eGFR) was 69.4(15.8-110.9) ml/min/1.73 m^2^. The study included 260 males (60.9%) and 167 females (39.1%). Of the patients, 44 (10.3%) had diabetes; 186 patients (43.6%) had hypertension. The average global PSQI score was 9.4 ± 2.5. A total of 332 patients (77.8%) had poor sleep quality as defined as a PSQI score > 5.

### Comparison of the clinical and laboratory data between the poor sleep quality group and the good sleep quality group

Compared with the good sleepers, the patients with poor sleep quality had an increased LVMI level (47.1 ± 14.6 g/m^2.7^ vs. 41.7 ± 9.5 g/m^2.7^, P < 0.05), a higher proportion of LVH (41.3% vs. 28.1%; *p* < 0.05), a lower E/A ratio (1.2 ± 0.4 vs. 1.3 ± 0.5, P <0.05), a higher BDI score (4 (2–8) vs. 1 (0–2); p < 0.001). The poor sleep quality group had a higher level of serum creatinine [114 (70–423) umol/L vs. 89 (66–214) umol/L, P <0.05] and a lower eGFR value [65.9 (13.2-109.6) ml/min/1.73 m^2^ VS. 85.3 (32.3-121.2) ml/min/1.73 m^2^, P <0.05]. The two subgroups were similar for the other variables. The results are shown in Table 1.

**Table 1:** Characteristics of the patients in the good sleepers group and the poor sleepers group

**Characteristic**	**Overall**	**Good sleepers**	**Poor sleepers**	**P value**
	**(n = 427)**	**(n = 95)**	**(n = 332)**	
Age (years)	39 ± 15	38 ± 13	39 ± 15	0.141
Sex ratio (M/F)	260/167	56/39	204/128	0.401
Duration of CKD (months)	5 (1–24)	6 (1–24)	5 (1–24)	0.761
Current smoker, n (%)	79 (18.5%)	20 (21.1%)	59 (17.8%)	0.457
Alcohol intake, n (%)	26 (6.1%)	7 (7.4%)	19 (5.7%)	0.626
BMI (kg/m^2^)	22.8 ± 3.6	22.7 ± 4.0	22.8 ± 3.7	0.784
ACEI/ARB (%)	240 (56.2)	56 (58.9)	184 (55.4)	0.560
Diabetes (%)	44 (10.3)	6 (6.3)	39 (11.7)	0.087
Proteinuria (g/24 h)	1.41 (0.46–3.82)	1.40 (0.46–4.12)	1.42 (0.46–3.76)	0.793
Hemoglobin (g/L)	118 ± 29	122 ± 25	117 ± 29	0.261
Albumin (g/L)	33.3 ± 8.9	33.0 ± 9.0	33.3 ± 8.9	0.810
Globulin( g/L)	23.5 ± 5.1	23.2 ± 5.2	23.5 ± 5.0	0.713
Serum cystatin C (mg/L)	1.5 (1.0-3.5)	1.4 (1.0-2.5)	1.4 (0.9-3.4)	0.450
Serum creatinine (μmol/L)	104 (69–355)	89 (66–214)	114 (70–423)	0.024
eGFR (ml/min/1.73 m^2^)	69.4 (15.8-110.9)	85.3 (32.3-121.2)	65.9 (13.2-109.6)	0.040
Cholesterol (mmol/L)	5.0 (4.0-6.8)	5.2 (4.2-6.6)	5.0 (4.0-7.1)	0.596
Triglycerides (mmol/L)	1.7 (1.1-2.6)	1.8 (1.1-2.9)	1.8 (1.1-2.7)	0.513
LDL-C (mmol/L)	3.1 (2.3-4.5)	3.2 (2.6-4.3)	3.1 (2.3-4.6)	0.426
HDL-C (mmol/L)	1.3 ± 0.5	1.2 ± 0.4	1.3 ± 0.5	0.932
Glucose (mmol/L)	4.7 (4.2-5.3)	4.6 (4.2-5.0)	4.7 (4.2-5.3)	0.391
Calcium (mg/dL)	9.2 ± 0.7	9.1 ± 0.7	9.2 ± 0.7	0.139
Phosphate (mmol/L)	1.4 ± 0.5	1.3 ± 0.4	1.4 ± 0.5	0.274
iPTH (pg/mL)	54.6 (32.1-150.2)	48.1 (32.0-83.2)	54..7 (31.1-153.0)	0.173
Serum uric acid (umol/L)	440 ± 142	441 ± 139	441 ± 143	0.967
LVEF (%)	66.9 ± 7.7	67.9 ± 6.7	66.7 ± 7.9	0.330
LVMI (g/m^2.7^)	46.2 ± 14.0	41.7 ± 9.5	47.1 ± 14.6	0.019
LVH (%)	158 (40.2)	18 (28.1)	140 (41.3)	0.011
E/A ratio	1.2 ± 0.4	1.3 ± 0.5	1.2 ± 0.4	0.027
CIMT (mm)	0.66 ± 0.20	0.64 ± 0.19	0.67 ± 0.20	0.443
Hypertension (%)	186 (43.6)	43 (45.3)	143 (43.1)	0.813
SBP (mmHg)	138.2 ± 22.2	137.9 ± 20.9	138.3 ± 22.5	0.921
DBP (mmHg)	84.8 ± 13.1	85.1 ± 11.0	84.7 ± 13.5	0.826
Depression (%)	176 (41.2)	12 (12.6)	164 (49.4)	<0.001
BDI-SF score	3 (1–7)	1 (0–2)	4 (2–8)	<0.001
PSQI score	9.4 ± 2.5	3.5 ± 1.3	10.9 ± 2.7	<0.001
CKD stage				
CKD(I- II) (%)	222	55 (24.8)	167 (75.2)	0.117
CKD (III- V) (%)	205	40 (19.5)	165 (80.5)	

(SBP: systolic blood pressure; DBP: diastolic blood pressure; BMI: body mass index, eGFR: estimated glomerular filtration rate; LDL-C: low-density lipoprotein cholesterol; HDL-C: high-density lipoprotein cholesterol; iPTH: intact parathyroid hormone; SBP: systolic blood pressure; DBP: diastolic blood pressure; BDI-SF: short form of the Beck Depression Inventory; CKD: Chronic kidney disease; CIMT: Carotid intima-media thickness; E/A ratio: Early/late diastolic peak flow velocity ratio; LVH: Left ventricular hypertrophy; LVMI: left ventricular mass index; Serum calcium is corrected by the following formula:[calcium] (mg/dL) = measured [calcium] + ( 4.0-[serum albumin(mg/dl)]) × 0.8, PSQI: Pittsburgh Sleep Quality Index).

### Independent characteristics of patients with poor sleep quality

Table 2 demonstrates the strengths of the associations between sleep quality and the clinical and laboratory characteristics. The univariate linear regression analysis revealed that older age, a longer duration of CKD, lower eGFR, lower hemoglobin level, higher BDI scores, higher serum levels of phosphate and iPTH were significantly associated with poor sleep quality. We found that only older age and higher BDI scores were independently associated with poor sleep quality in the multivariate regression analysis.

**Table 2:** Univariate and multivariate Linear Regression model for predictors of sleep quality (PSQI scores)

	**Standardized coefficient β**	**Crude P value**	**Adjusted standardized coefficient β**	**AdjustedP value**^**b**^
Age (years)	0.149	0.004	0.155	0.004
Duration of CKD (months)	0.104	0.054	-	-
Male vs. female	0.024	0.637	-	-
Current smoker (no vs. yes)	-0.046	0.283		
Alcohol intake (no vs. yes)	-0.039	0.368		
BMI (kg/m^2^)	0.010	0.863		
Diabetes (no vs. yes)	0.086	0.116	-	-
Proteinuria (g/24 h)	-0.047	0.398		
Hemoglobin (g/L)	-0.174	0.004	-	-
Albumin (g/L)	0.065	0.224		
eGFR (ml/min/1.73 m^2^)	-0.224	<0.001	-	-
Cholesterol (mmol/L)	-0.031	0.575		
Triglyceride (mmol/L)	-0.033	0.543		
LDL-C (mmol/L)	-0.054	0.339		
Calcium (mg/dL)	0.073	0.152		
Phosphate (mmol/L)	0.140	0.007	-	-
iPTH (pg/mL)	0.139	0.018	-	-
Serum uric acid (umol/L)	0.029	0.587		
SBP (mmHg)	0.030	0.585		
DBP (mmHg)	0.064	0.218		
BDI-SF score	0.413	<0.001	0.427	<0.001

(^a^all of the variables were adjusted by age, sex. ^b^The age, sex, duration of CKD, diabetic vs. non diabetic status, eGFR, hemoglobin, phosphate, iPTH and BDI-score were included in the multivariate model (stepwise method). -Without significance and not able to be included in the multivariate model. eGFR: estimated glomerular filtration rate; iPTH: intact parathyroid hormone, SBP: systolic blood pressure; DBP: diastolic blood pressure; BDI-SF: short form of the Beck Depression Inventory).

### Association of sleep quality with LVH

**Figure 1:**
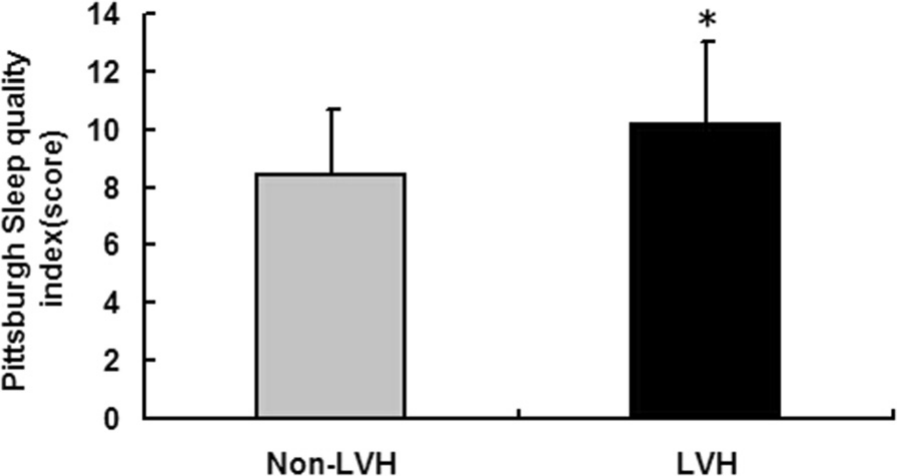
Comparison of the PSQI score between the Non-LVH group and the LVH group. * means P < 0.001.

Figure 1 shows that the LVH patients had higher PSQI scores when compared with the non-LVH group (P < 0.001). Table 3 shows the associations of LVH and the demographic and clinical factors. Age; the levels of hemoglobin, serum calcium, phosphate and iPTH; Serum uric acid; eGFR < 60 ml/min/1.73 m^2^; SBP and DBP; and poor sleep quality were significantly associated with LVH when adjusted only for age and gender. In the multivariate regression analysis, an eGFR < 60 ml/min/1.73 m^2^, a higher serum iPTH level, higher SBP and poor sleep quality were independently associated with LVH.

**Table 3:** Univariate and multivariate logistic regression analysis for left ventricular hypertrophy (1 = no LVH; 2 = LVH)

	**OR(95% CI)**	**Crude**	**Adjusted**	**Adjusted**
		**P value**^**a**^	**OR(95% CI)**	**P value**^**b**^
Age (per 1 year)	1.061 (1.040-1.082)	<0.001	-	-
Duration of CKD (per 1 month)	0.999 (0.993-1.005)	0.759	-	-
Male vs. female	1.525 (0.879-2.645)	0.133	-	-
Current smoker (no vs. yes)	0.931 (0.476-1.822)	0.835		
Alcohol intake (no vs. yes)	1.980 (0.780-5.027)	0.151		
BMI (per 1 kg/m^2^)	0.992 (0.908-1.083)	0.855		
Diabetes (no vs. yes)	1.203 (0.614-2.989)	0.690		
ACEI/ARB (no/yes)	0.768 (0.431-1.368)	0.370		
Proteinuria (per 1 g/24 h)	0.939 (0.815-1.081)	0.381		
Hemoglobin (per 1 g/L)	0.971 (0.960-0.983)	<0.001	-	-
ALB (per 1 g/L)	1.017 (0.977-1.061)	0.421		
eGFR < 60 ml/min/1.73 m^2^	8.150 (3.234-20.542)	<0.001	3.943 (1.255-12.387)	0.019
Cholesterol (per 1 mmol/L)	0.940 (0.844-1.048)	0.264		
Triglyceride (per 1 mmol/L)	0.930 (0.711-1.217)	0.598		
LDL-C (per 1 mmol/L)	0.936 (0.816-1.047)	0.347		
Calcium (per 1 mg/dL)	0.397 (0.223-0.646)	<0.001	-	-
Phosphate (per 1 mg/dL)	2.998 (1.500-5.992)	0.002	-	-
iPTH (per 1 pg/mL)	1.006 (1.003-1.010)	<0.001	1.004 (1.002-1.007)	0.001
Serum uric acid (per 1 umol/L)	1.003 (1.001-1.006)	0.004	-	-
PSQI score	1.117 (1.041-1.200)	0.002	1.092 (1.011-1.179)	0.025
BDI-SF score	1.061 (0.012 - 1.111)	0.014	-	-
SBP (per 1 mmHg)	1.044 (1.028-1.061)	<0.001	1.034 (1.015-1.054)	<0.001
DBP (per 1 mmHg)	1.056 (1.032-1.081)	<0.001	-	-

(^a^all of the variables were adjusted by age, sex. ^b^The age, sex, hemoglobin, eGFR, phosphate, serum calcium, Serum uric acid, iPTH, SBP, DBP, BDI -score and PSQI score were included in the multivariate model (backward method). -Without significance and not able to be included in the multivariate model. eGFR: the estimated glomerular filtration rate; iPTH: intact parathyroid hormone, SBP: systolic blood pressure; DBP: diastolic blood pressure; PSQI: Pittsburgh Sleep Quality Index).

### Association of sleep quality with the E/A ratio

**Figure 2:**
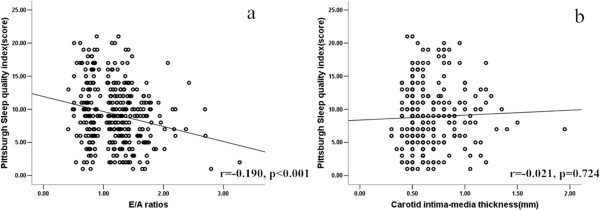
Correlation analysis between the PSQI score and the E/A ratios (a) and CIMT(b).

Figure 2 shows that the PSQI score was significantly associated with the E/A ratio (r = 0.190, p < 0.001) and was not associated with the CIMT (r = -0.021, p = 0.724). As shown in Table 4, the linear regression analyses revealed that age, the serum phosphate, BMI, Serum uric acid, SBP, DBP and PSQI scores were negatively associated with the E/A ratio, whereas the eGFR was positively associated with the E/A ratio when adjusted only with age and gender. We found that age, the DBP, BMI and PSQI scores were independent factors associated with the E/A ratio in the further multivariate regression analysis.

**Table 4:** Univariate and multivariate Linear Regression model for the predictors of the E/A ratios

		**P value**^**a**^	**Coefficient β**	**P value**^**b**^
Age (years)	-0.650	<0.001	-0.613	<0.001
Duration of CKD (months)	-0.059	0.186		
Male vs. female	-0.032	0.459	-	-
Current smoker (no vs. yes)	-0.054	0.318		
Alcohol intake (no vs. yes)	-0.024	0.585		
BMI (kg/m^2^)	-0.139	0.002	-0.113	0.029
ACEI/ARB (no/yes)	0.041	0.369		
Diabetes (no vs. yes)	-0.060	0.192		
Proteinuria (g/24 h)	-0.005	0.907		
Hemoglobin (g/L)	0.008	0.870		
Albumin (g/L)	-0.031	0.485		
eGFR (ml/min/1.73 m^2^)	0.126	0.012	-	-
Cholesterol (mmol/L)	-0.048	0.286		
Triglyceride (mmol/L)	-0.060	0.168		
LDL-C (mmol/L)	-0.053	0.247		
Calcium (mg/dL)	-0.064	0.138	-	-
Phosphate (mmol/L)	-0.096	0.025	-	-
iPTH (pg/mL)	-0.068	0.140	-	-
Serum uric acid (umol/L)	-0.168	<0.001	-	-
PSQI scores	-0.129	0.007	-0.115	0.028
BDI-SF score	-0.065	0.229	-	-
SBP (mmHg)	-0.173	<0.001	-	-
DBP (mmHg)	-0.191	<0.001	-0.184	<0.001

(^a^all of the variables were adjusted by age, sex. ^b^the age, sex, BMI, eGFR, serum calcium, phosphate and iPTH, Serum uric acid, SBP, DBP, BDI score and PSQI scores were included in the multivariate model (stepwise method). -Without significance and not able to be included in the multivariate model and hence these independent variables were not included into the model to predict dependent variable. DBP: diastolic blood pressure; eGFR: estimated glomerular filtration rate; iPTH: intact parathyroid hormone, SBP: systolic blood pressure; PSQI: Pittsburgh Sleep Quality Index).

## Discussion

Few studies have addressed sleep quality in subjects with CKD prior to ESRD. Our study first investigated sleep quality in Chinese CKD patients prior to dialysis and found that the prevalence of poor sleep quality was as high as 77.8%. Previous studies have shown that the prevalence of sleep disorders based on questionnaires have provided widely different estimates of in-patients with CKD, which ranged from 14–85% in CKD patients [3], 41–83% in HD patients [21-23] and 49–85% in PD patients [7,21,24]. These rates might vary be because of the different life styles, genetic factors, environmental conditions and primary cause of CKD in the study populations or because different questionnaires were used to estimate sleep quality in the studies. Our study shows that a higher sleep quality score was positively associated with older age, higher phosphate levels, iPTH and a longer duration of CKD and inversely associated with the hemoglobin level and the eGFR in the univariate analyses. After adjusting for the confounding factors, only higher BDI scores and older age were independently associated with sleep quality. Numerous factors could potentially contribute to the high prevalence of sleep problems in CKD patients including older age, pain, depression, dyspnea, nausea and pruritus; however, it appears that there is no association between sleep quality and specific laboratory variables such as hemoglobin, albumin, calcium or phosphorus in CKD patients [4,25]. As in most of the studies [4,9,26], our study failed to show a relationship between renal function and sleep quality in the multivariate linear regression analysis, suggested that poor sleep quality could exist at a very early stage in CKD and that the decreased renal function and other specific laboratory variables might not be the direct cause of poor sleep in CKD patients. A series of associated comorbidities and symptoms such as high depression scores, pain, nausea and itchiness could be aggravated by declining renal function and affect the sleep quality in CKD patients [4]. In research with a small sample size (n = 58), Kurella et al. [8] reported a significant association between eGFR and sleep quality among non-African-American subjects.

Previous studies have shown that poor sleep quality is an important risk factor for the development of cardiovascular diseases [5] in the general population. The PSQI score was reported to be positively associated with cardiovascular disease in CAPD patients [7]. Recent research indicated that an increase in observed/predicted LVM is independently associated with adverse cardiovascular outcomes in patients with stages 3–5 CKD [27]. Diastolic dysfunction is a strong predictor of mortality in patients with CKD [28]. From the univariate and multivariate regression analysis, our study first showed that higher PSQI scores were associated with LVH and lower E/A ratios. The mechanisms of these associations are unclear. One of the explanations might be that poor sleep quality is associated with blunting of the nocturnal dip in blood pressure and with higher activation of the sympathetic nervous system [29]; the sympathetic nervous system and non-dipping blood pressure are risk factors of LVH and lower E/A ratios [30,31]. Reduced sleep quality might decrease the normal nocturnal increase in the plasma renin activity and aldosterone levels [3] and might lead to LVH. Our findings might facilitate a better understanding of the pathogenesis and consequences of cardiovascular diseases in CKD patients with poor sleep quality.

The strength of our study was that we were the first to report the prevalence and risk factors of poor sleep quality in Chinese pre-dialysis CKD patients; we first reported that poor sleep quality is closely related with cardiovascular damage. Several limitations should be considered when interpreting our findings. First, the subjective reporting of sleep quality by use of the PSQI might lead to reporting bias. It has been shown that patients with sleep problems are more likely to overestimate their sleep difficulties [32]. In addition, the PSQI measures overall general sleep quality rather than a specific form of sleep disorder. Future studies with objective measures are warranted, but using a polysomnogram or other objective measure is costly in a large clinical study such as this study. Second, the nature of the cross-sectional design in this study did not allow us to determine a causal relationship between poor sleep quality and cardiovascular damage and pre-dialysis patients. Cardiovascular damage might lead to poor sleep quality [33,34]. Further multi-center follow-up studies with larger samples are necessary to determine the effect of factors of such as poor sleep quality and to investigate the complex nature of the relationship between sleep quality and cardiovascular damage in Chinese CKD patients. In addition, although our study excluded sleep apnea and chronic obstructive pulmonary disease and find that depression can affect sleep quality, other factors, such as pruritis, chronic pain and caffeine use might affect sleep, were not include in this study. These may also confound the results. Finally, the studied population in this study was relatively young. These findings in the current study might not be able to generalize to older population.

## Conclusions

This study demonstrates a high prevalence of poor sleep quality in pre-dialysis CKD patients. Poor sleep quality is an independent risk factor for LVH and ventricular diastolic function in pre-dialysis CKD patients. Assessment with the PSQI is the first step in detecting sleep disorders and should be used in all renal patients to determine for whom more accurate procedures are needed to identify and treat specific disorders. Treatment to improve sleep quality might improve the overall quality of life and positively affect CVD.

## Competing interests

No financial or other conflicts of interest are declared by the authors.

## Authors’ contributions

Conceived and designed the experiments: JZ and TL. Performed the experiments: CW, WG, YT, CL, WZ and ZY. Analyzed the data: JZ and CW. Contributed reagents/materials/analysis tools: HP and ZY. Wrote the paper: JZ. Review the manuscript for intellectual content: Dr CW, JZ and TL. All authors read and approved the final manuscript.

## Pre-publication history

The pre-publication history for this paper can be accessed here:

http://www.biomedcentral.com/1471-2369/15/131/prepub
